# Type 2 inflammation and biological therapies in asthma: Targeted medicine taking flight

**DOI:** 10.1084/jem.20221212

**Published:** 2023-06-02

**Authors:** Imran Howell, Aleksandra Howell, Ian D. Pavord

**Affiliations:** 1 Nuffield Department of Medicine, Respiratory Medicine Unit, NIHR Respiratory Biomedical Research Centre, University of Oxford, Oxford, UK

## Abstract

The field of asthma has undergone a dramatic change in recent years. Advances in our understanding of type 2 airway inflammation have driven the discovery of monoclonal antibodies targeting specific aspects of the immune pathway. In landmark trials, these drugs have shown efficacy in reducing asthma attacks and exposure to oral corticosteroids, important causes of morbidity in people with asthma. Our review explores the key features of type 2 inflammation in asthma and summarizes the clinical trial evidence of the novel monoclonal antibody treatments and future avenues for treatment.

## Teaser

In this Review, we discuss the key features of type 2 inflammation in asthma, summarize the clinical trial evidence of monoclonal antibody asthma treatments, and explore future avenues for targeted asthma treatment.

## Introduction

Asthma is characterized by variable symptoms of breathlessness, cough, chest tightness, and wheeze. Diagnosis is supported by evidence of reversible airflow obstruction on spirometry or airway hyperresponsiveness through bronchial provocation testing (Reddel et al., 2022). Asthma is acknowledged to be a heterogeneous disorder in relation to the mechanisms that drive morbidity. The treatable traits framework has emerged as a useful approach to untangle contributory factors and target the right treatment for the right patient (Melhorn et al., 2022). The most important treatable trait is the presence of raised type 2 airway inflammation (type 2-high). This is characterized by eosinophilic airway inflammation and makes an important contribution to clinical problems in over 50% of patients with asthma (Frøssing et al., 2021). Active eosinophilic airway inflammation is associated with recurrent asthma attacks, also termed exacerbations, irreversible lung function decline, and “people remodeling” as a result of type 2 inflammation in other sites and the side effects of treatment with oral corticosteroids (OCS). Biomarkers, including blood eosinophils, fraction exhaled nitric oxide (FeNO), and, to a lesser extent, serum IgE and allergy tests are useful surrogate measures of type 2 inflammation (Pavord et al., 2017).

For over 30 yr, the cornerstone of asthma treatment has been inhaled corticosteroids (ICSs). ICSs reduce eosinophilic airway inflammation and are proven to improve symptoms and reduce asthma attacks (Beasley et al., 2019). However, around 10% of adults and 2.5% of children respond poorly to this treatment and have severe asthma (Settipane et al., 2019). Severe asthma should be distinguished from difficult asthma, where poor control is a function of poor treatment adherence or the presence of comorbid factors (Wenzel, 2021). Patients with severe asthma are frequently treated with numerous courses of high-dose OCS to quench type 2 airway inflammation. However, the permanent and temporary adverse effects of OCS are abundant and serious (Price et al., 2018). Over the last 20 yr, biomarker-directed biological therapies have revolutionized severe asthma. Biologics target specific aspects of type 2 inflammatory pathway and are broadly shown to reduce asthma attacks and reliance on OCS treatment. Given the rapidly shifting approach to type 2-high asthma, it is important to understand the biological and clinical basis for asthma biologics. Our review will examine the fundamental aspects of type 2 inflammation in asthma and the evidence for biologic asthma treatments.

## Type 2 inflammation in asthma

Our understanding of type 2 immunity has matured from simply a tool to regulate type 1 immunity to a more complex role including host defense and tissue repair (Wynn, 2015). The key evolutionary role of type 2 immunity is as a parasite defense and wound healing mechanism, where it has advantages over a purely type 1 response (Gause et al., 2013).

**Figure 1.**
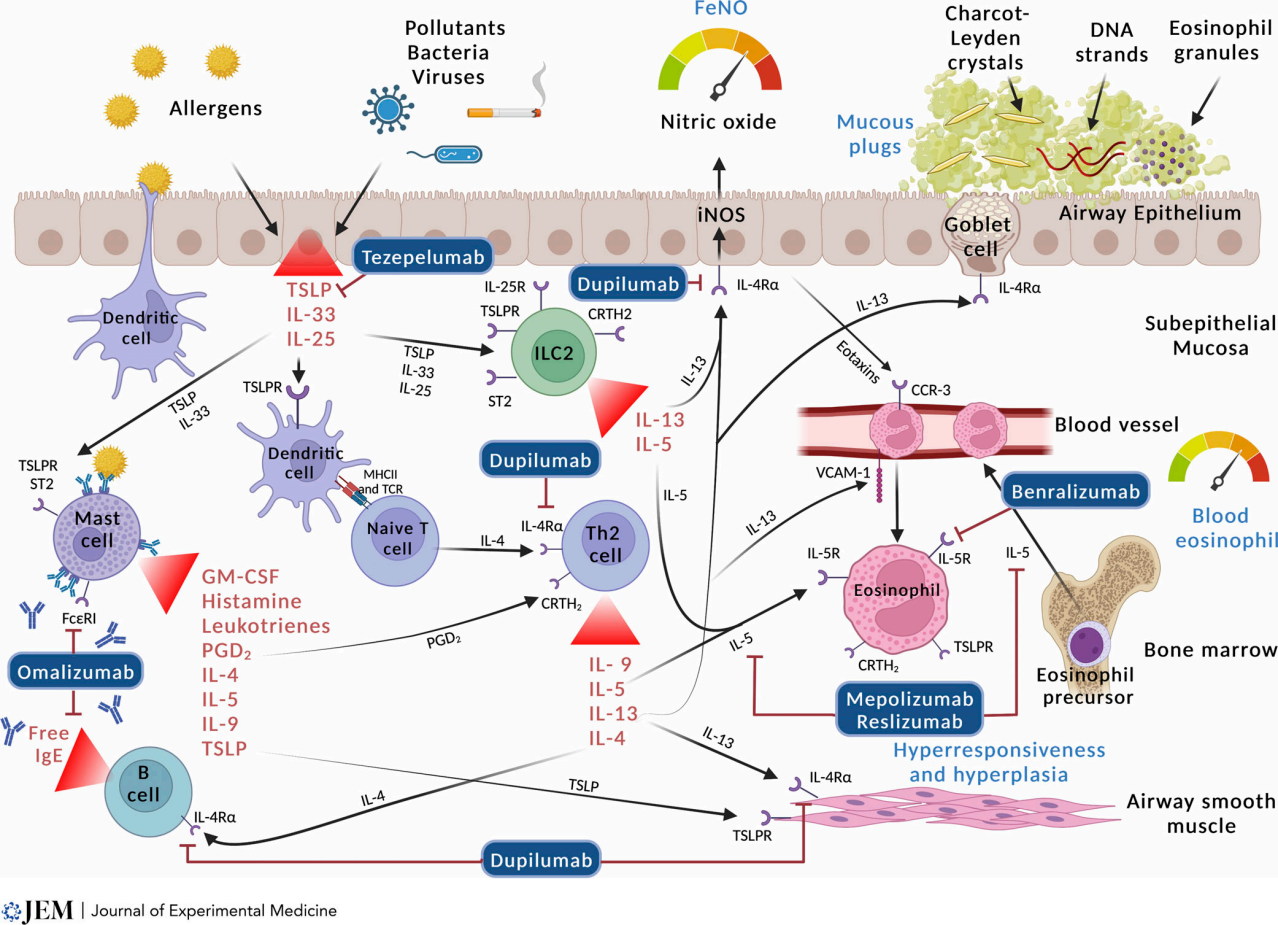
**Type 2 inflammation in asthma and targets for monoclonal antibody treatment.** Type 2-high inflammation is the predominant treatable trait in asthma. It is present in both childhood-onset allergic asthma and adult-onset eosinophilic asthma. Different people have different pathways that predominate, and our understanding of why these develop and how to best characterize their activity is developing. In allergen-sensitized people, DCs present inhaled aeroallergens to tissue-resident CD4^+^ type 2 helper T lymphocytes (Th2 lymphocytes). These produce a large amount of type 2 cytokines (IL-4, IL-5, IL-13, and IL-9). In parallel, alarmins also stimulate the Th2 lymphocyte pathway, as well as ILC2, to produce type 2 cytokines. IL-4 and IL-13 stimulate B cells to mature into plasma cells and secrete IgE. IgE binds to the high-affinity FcεRI on mast cells, leading them to release PGD2 and histamine amongst other cytokines causing bronchoconstriction. Additionally, IL-13 induces the production of nitric oxide via the iNOS enzyme. This process can be measured by FeNO. IL-13 also stimulates mucous hypersecretion and smooth muscle contraction, and both IL-4 and IL-13 facilitate the recruitment of eosinophils from circulation to the airway mucosa. IL-5 enhances the proliferation and activation of eosinophils which degranulate causing tissue damage and producing mucous plugs through the formation of CLCs, eosinophil granules, and eosinophil extracellular traps.

The asthmatic airway is characterized by chronic inflammation of the airway wall due to a complex infiltration and interplay of immune cells including eosinophils, neutrophils, lymphocytes, dendritic cells (DCs), innate lymphoid cells (ILCs), and mast cells. The inflammation leads to narrowing of the airway, airway hyperresponsiveness, airway mucous plugging, and airway remodeling. This pathology underpins the typical asthma symptoms as well as the airflow obstruction seen on spirometry (Hammad and Lambrecht, 2021). See Fig. 1 for a graphic representation of the type 2-high pathways in asthma.

### Sounding the alarm

Airway epithelial cells are the interface between the external environment and internal structures. They act as the primary line of defense against pathogens and other inhaled insults such as allergens or chemical irritants (Mitchell and O’Byrne, 2017). This defense is orchestrated by the release of epithelial cytokines (alarmins) in response to epithelial damage through activation of toll-like receptors or protease-activated receptors. The three best-studied alarmins are IL-33, thymic stromal lymphopoietin (TSLP), and IL-25. Receptors for these alarmins are expressed on a wide range of cells including DCs, eosinophils, basophils, mast cells, group 2 ILCs (ILC2), and macrophages (Whetstone et al., 2022). TSLP mRNA expression is elevated in epithelial cells and correlates with airway obstruction (Semlali et al., 2010). IL-25 and IL-33 are raised in type 2-high asthma and are associated with airway hyperresponsiveness and disease severity (Ballantyne et al., 2007; Wang et al., 2018).

TSLP, IL-33, and IL-25 stimulate ILC2 and Th2 cells to produce type 2 cytokines (Halim et al., 2018; Huang et al., 2015; Toki et al., 2020). ILC2 cells have been shown in mouse models to be a key intermediary in the processes leading to tissue eosinophilia, goblet cell metaplasia, and bronchial hyperreactivity (Lambrecht et al., 2019). Once stimulated by alarmins, ILC2s produce IL-5, IL-9, and IL-13, and act in a complementary way to Th2 cells (Gurram and Zhu, 2019). ILC2s may have a role in the pathogenesis of viral-induced asthma exacerbations. In a human rhinovirus challenge study, an ILC2-predominant inflammatory profile in patients with asthma was associated with increased severity and duration of rhinovirus infection compared with healthy subjects (Dhariwal et al., 2021). ILC2s in human asthma remain an area of active research, with the main issue being the extent that ILC2s and Th2 cells overlap in their function.

TSLP and IL-33 may also encourage proliferation of eosinophilic progenitor cells, attenuate eosinophil apoptosis, and promote eosinophil migration and tissue accumulation by upregulating intracellular cell adhesion molecule-1 (ICAM-1) and CD18 and suppressing L-selectin (Smith et al., 2015; Wang et al., 2018; Wong et al., 2010). TSLP, IL-33, and IL-25 stimulate basophils to release type 2 cytokines, histamine, and leukotrienes (Salter et al., 2016; Salter et al., 2015). TSLP enhances mast cell production of type 2 cytokines and chemokines, which can be perpetuated by the mast cells themselves producing more TSLP in the presence of IL-4 or IgE (Kaur et al., 2012; Okayama et al., 2009). Alarmins also interact with DCs, causing them to migrate from the lung tissue to the draining lymph nodes to induce CD4^+^ T cells to become effector Th2 cells, which then produce type 2 cytokines (Froidure et al., 2014; Ito et al., 2005; Watanabe et al., 2004).

### The allergic cascade

Th2 cells formed in the draining lymph nodes also stimulate B cells to mature into plasma cells and produce antibodies (Dullaers et al., 2017). IL-4 and IL-13 encourage the production of IgE which in turn binds to the high-affinity FcεRI expressed on mast cells, basophils, eosinophils, DCs, airway smooth muscle cells, and epithelial cells (Froidure et al., 2016).

Allergic asthma is typically early onset but can persist into adulthood. DCs present inhaled aeroallergens, such as house dust mite, animal dander, or pollen, to tissue-resident memory Th2 lymphocytes, expressing antigen-specific TCRs (Gill, 2012; Plantinga et al., 2013). IgE crosslinking by an allergen causes mast cells and basophils to release histamine, tryptase, cysteinyl leukotrienes, prostaglandin D2 (PGD2), and type 2 cytokines (Galli and Tsai, 2012). Mast cells migrate to the submucosa and airway smooth muscle where PGD2 and histamine interact with PGD2 receptors (DP1 and CRTH2; Pettipher et al., 2007). This rapid process physiologically leads to vascular permeability, mucous hyperproduction by goblet cells, and airway hyperresponsiveness due to smooth muscle contraction and hypertrophy. In a vicious cycle, IgE also facilitates allergen presentation to memory Th2 cells thereby reducing the threshold for future, similar responses (Mojtabavi et al., 2002).

### The magnetism of IL-4 and IL-13

IL-4 and IL-13 play a powerful and intricate role in the type 2 cytokine response due to their effect on nearly every cell relevant to asthma. There are four receptor chains that compose the IL-4R complexes: IL-4Rα, γ chain, IL-13Rα1, and IL-13Rα2 (Gour and Wills-Karp, 2015). On naive lymphocytes, IL-4 binds to the heterodimer of IL-4Rα and the γ chain (type 1 IL-4R) to help stimulate differentiation into Th2 cells, maintain this Th2 response through autocrine effects, and facilitate IgE and IgG1 synthesis by B cells (Cohn et al., 1997). This pathway is essential in the development of allergic asthma and is likely genetically controlled, evidenced by a polymorphism in the IL-4, IL-13, and RAD50 genes associated with asthma and atopy (Demenais et al., 2018).

On airway epithelial and smooth muscle cells, IL-4 and IL-13 bind to the heterodimer IL-4Rα and IL-13Rα1 (type 2 IL-4R; Kuperman and Schleimer, 2008). This leads to goblet cell metaplasia, myofibroblast metaplasia, smooth muscle hyperresponsiveness, and production of inducible nitric oxide synthase (iNOS; McKnight et al., 2020). Mucous hypersecretion, airway remodeling, airway eosinophilia, and airway hyperresponsiveness—with modulation through iNOS-induced bronchodilation (Ricciardolo, 2003)—are therefore critically dependent on IL-4 and IL-13.

Furthermore, IL-4 and IL-13 promote the expression of vascular cell adhesion molecule-1 (VCAM-1), ICAM-1, and the generation of eosinophil-attracting chemokines, such as eotaxins (Conroy and Williams, 2001; Doucet et al., 1998; Nakajima et al., 1994). In this way, IL-4 and IL-13 coordinate the extravasation of eosinophils and their trafficking to the airway epithelium through the epithelial cell wall. This process is analogous to a magnet, and its activity is reflected by the measurement of FeNO (Couillard et al., 2022).

All the IL-4R signaling pathways operate in a JAK-STAT6-dependent manner (Georas et al., 2021). The role of the homodimer IL-13Rα2 is less clear but, in its soluble form, is likely a decoy receptor for IL-13 to modulate the intensity of type 2 IL-4R signaling (Gour and Wills-Karp, 2015).

### Arming the bombs: Eosinophils in asthma

Eosinophils develop in the bone marrow from pluripotent CD34^+^ stem cells. Expression of certain transcription factors induces eosinophil differentiation (C/EPB, GATA-1, PU.1). IL-5, GM-CSF, and IL-3 synergistically continue this process of differentiation and maturation in the bone marrow (Du et al., 2002). IL-5 is the most important cytokine for maturing eosinophils and mobilizing them from the bone marrow (Collins et al., 1995). IL-5 is released by Th2 cells and ILC2s following allergic or noxious stimuli. IL-5 binds to eosinophils via an IL-5–specific α-subunit (IL-5Rα) and a β-subunit common to GM-CSF and IL-3. Eosinophils also possess receptors for IL-4, IL-13, IL-33, TSLP, and TGF-β (Matucci et al., 2019).

In an asthmatic person, eosinophils are drawn to the lungs by eotaxins (CCL-11, CCL-24, and CCL-26) that bind to the CCR3 receptors on eosinophils and induce chemotaxis (Provost et al., 2013). PGD2 and CRTH2 also play a role in enhancing the chemoattractant effects of eotaxins (Fajt et al., 2013; Monneret et al., 2001). Eosinophils adhere to and roll along the vascular endothelium with P-selectin binding to P-selectin glycoprotein ligand-1 on the endothelium (Rosenberg et al., 2007). This is potentiated by IL-13 (Woltmann et al., 2000). The eosinophil extravasates using VLA-4 integrin that binds to VCAM-1.

Once in the airway tissue, eosinophils can generate inflammation in a variety of ways. By degranulating, akin to detonating a bomb, eosinophils damage lung structural cells through the release of cytotoxic proteins including major basic protein (MBP), eosinophil peroxidase (EPO), eosinophil-derived neurotoxin (EDN), and eosinophil cationic protein (ECP; Cañas et al., 2018). Proinflammatory cytokines (TNFα, IL-1β, IL-6, and IL-8) and lipid mediators (leukotrienes), mast cells, and Th2 cytokines are also released leading to bronchial hyperresponsiveness, mucous hypersecretion, and perpetuation of the inflammatory cascade (Blanchard and Rothenberg, 2009). Eosinophilic granule content near airway nerves can also change parasympathetic and sensory nerve tone (Drake et al., 2018). Persistent cell damage ultimately triggers repair pathways that cause airway remodeling via fibroblast proliferation, smooth muscle hypertrophy, and angiogenesis (Chen et al., 2021; Gomes et al., 2005; Halwani et al., 2013). Airway remodeling is a complex process, beyond the scope of this article, but is inherently linked with the severity and chronicity of type 2 airway inflammation (Hough et al., 2020). It is an important cause of persistent airflow obstruction—defined as airflow obstruction on spirometry that does not improve with β-2 agonist treatment.

Another cause of persistent airflow obstruction is mucous plugging. Eosinophils can form extracellular traps (EETs) made from extracellular DNA fibers (Ueki et al., 2016). Functionally, these are antimicrobial; however, in asthma they combine with Charcot–Leiden crystals to form more viscous mucous that is harder to expectorate (Persson et al., 2019). The abundant mucous plugging in type 2-high asthma contributes to the non-bronchodilator responsive airway obstruction seen on spirometry (Dunican et al., 2018).

## Biological therapies: Averting a Pyrrhic victory

In 1956, OCSs were shown to be effective in treating asthma attacks (MRC, 1956). Trials between 1983 and 1993 confirmed their clinical efficacy versus placebo in terms of symptom recovery from an asthma attack and preventing relapse (Rowe et al., 2007).

ICSs have a much wider therapeutic index and are an important part of modern asthma treatment. They downregulate transcription of a wide range of cytokines, resulting in a reduction in Th2 cells, mast cells, macrophages, and DCs. Eosinophils are reduced in the airway epithelium because of apoptosis and by decreased chemotactic pull by chemokines such as eotaxin, which are also downregulated by ICSs. This effect is reflected by a reduction in FeNO (Heaney et al., 2019). However, around 10% of adults with unchecked type 2 inflammation do not respond to ICS leading to frequent rescue treatment or maintenance OCS. The cumulative OCS side effect burden in these people becomes intolerable. Asthmatic patients will endure bone fractures, mental health disorders, diabetes, and cardiovascular disease to rectify their breathing difficulties.

Biological therapies offer a reprieve by targeting specific parts of the type 2 inflammatory pathway. They have also proved safe and effective at reducing asthma attacks and oral steroid use. For clinical relevance and brevity, we will mainly focus on the mechanisms and efficacy of treatments that are currently licensed in adults. The key classes of monoclonal antibody are anti-IgE (omalizumab), anti-IL5/anti-IL5R (mepolizumab, reslizumab, and benralizumab), anti-IL4R (dupilumab), and anti-TSLP (tezepelumab; see Table 1).

**Table 1.** Characteristics of approved monoclonal antibody treatments for severe eosinophilic asthma

Characteristic	Omalizumab	Mepolizumab	Reslizumab	Benralizumab	Dupilumab	Tezepelumab
Mechanism	Anti-IgE	Anti-IL5	Anti-IL5	Anti-IL5Rα	Anti-IL4Rα	Anti-TSLP
Indication	Severe asthma	Severe asthma	Severe asthma	Severe asthma	Severe asthma	Severe asthma
Drug form	Prefilled syringe	Prefilled syringe, autoinjector pen	Intravenous infusion	Prefilled syringe, autoinjector pen	Prefilled syringe, autoinjector pen	Prefilled syringe
Licensed patient age	≥6 yr	≥6 yr	≥18 yr	≥12 yr	≥6 yr	≥12 yr
Safety concerns	Injection site reactions (2–10%), hypersensitivity reactions (<1%)	Injection site reactions (2–10%), hypersensitivity reactions (<1%), shingles (rare), helminth infection (rare)	Injection site reactions (2–10%), hypersensitivity reactions (<1%), helminth infection (rare)	Injection site reactions (2–10%), hypersensitivity reactions (<1%), helminth infection (rare)	Injection site reactions (2–10%), hypersensitivity reactions (<1%), hypereosinophilia (3%), conjunctivitis, helminth infection (rare)	Injection site reactions (2–10%), hypersensitivity reactions (<1%), pharyngitis, arthralgia, back pain
Typical patient group	Allergic asthma, childhood onset	Eosinophilic asthma, adulthood onset	Eosinophilic asthma, adulthood onset, polysorbate allergy	Eosinophilic asthma, adulthood onset	Eosinophilic or allergic asthma, childhood or adulthood onset	Eosinophilic or allergic asthma, adulthood onset, type 2 low asthma
Key biomarker(s) for response	Serum IgE (for dosing)	Raised blood eosinophils	Raised blood eosinophils	Raised blood eosinophils	Raised blood eosinophils and FeNO	Raised blood eosinophils and FeNO
Co-existing conditions treated by biologic	Chronic spontaneous urticaria, allergic rhino-conjunctivitis	ABPA, EGPA, chronic rhinosinusitis with nasal polyposis	Chronic rhinosinusitis with nasal polyposis	Chronic rhinosinusitis with nasal polyposis	Chronic rhinosinusitis with nasal polyposis, atopic dermatitis (eczema)	Chronic rhinosinusitis with nasal polyposis
Effect on blood eosinophil	↓	↓↓	↓↓	↓↓	↑ (returns to baseline after 1 yr)	↓
Effect on FeNO	↓	None	None	None	↓↓	↓↓
Effect on serum IgE	None	None	None	None	↓	↓
Effect on exacerbations	↓	↓↓	↓↓	↓↓	↓↓	↓↓
Effect on FEV1	None	↑	↑	↑	↑↑	↑↑
Oral steroid sparing	No	Yes	Yes	Yes	Yes	No (trial ongoing)

EGPA, eosinophilic granulomatosis with polyangiitis; ABPA, allergic bronchopulmonary aspergillosis; FeNO, fraction exhaled nitric oxide.

### Anti-IgE treatment

Omalizumab was the first monoclonal antibody approved for use in asthma. The drug binds to the Fc fragment of circulating IgE and prevents IgE binding to high-affinity receptors (FcεRI) on mast cells and basophils. This reduces cellular activation and inflammatory mediator release, particularly in the context of an allergic asthmatic response (Fahy et al., 1997). Omalizumab is approved for severe asthma in patients aged 6 yr or older with uncontrolled asthma, elevated total IgE, and sensitization to a perennial aeroallergen by skin-prick test or serum-specific IgE.

The efficacy of omalizumab is extensively studied in allergic asthma. A recent systematic review and meta-analysis of five adult studies found a 37% (CI 21–50%) absolute risk reduction in the annual exacerbation rate (AER; Henriksen et al., 2020). This was above the predefined minimally clinically important difference (MCID) of 25%. The studies were heterogeneous in terms of their follow-up length, the intensity of baseline asthma therapy, and number of previous exacerbations. The OCS sparing efficacy of omalizumab is unclear with only one small, unblinded study reporting a 45% reduction in OCS consumption (Siergiejko et al., 2011). There is no clear evidence that omalizumab improves lung function or asthma control and quality of life scores, as improvements in these were small and below the MCID. No study has shown a biomarker that effectively predicts response to omalizumab treatment. There is some observational evidence that omalizumab can be safely withdrawn after 2 yr of treatment (Domingo et al., 2018). Omalizumab has the most real-world pregnancy safety data of all the biologics and there is no signal of any congenital problems after 14 yr of treatment (Namazy et al., 2020).

Due to the inconsistent clinical evidence and unpredictable real-world data, omalizumab is best suited to a small, specific group of young asthmatics where allergy is the primary factor in their exacerbations. IgE appears to be the product of type 2-high inflammatory activation, rather than a key mediator.

### Anti-IL5 and anti-IL5Rα treatment

IL-5 plays a more central role in the pathogenesis of type 2-high asthma. Mepolizumab and reslizumab bind to IL-5, while benralizumab binds to IL-5Rα. All three treatments result in a marked reduction in blood eosinophils and are licensed for patients with severe eosinophilic asthma with a blood eosinophil count of at least 0.15 × 10^9^ cells per liter.

The path to success for anti-IL5 biologics was not straightforward (Pavord et al., 2021). Early clinical trials of mepolizumab in unselected asthma patients did not show a positive clinical effect on lung function despite evidence of a reduction in blood and sputum eosinophils. These trials overlooked the importance of selecting patients with evidence of eosinophilic airway inflammation and choosing a closely associated endpoint—asthma attacks. It took small, investigator-led trials targeting patients with raised blood eosinophils to demonstrate a large reduction in asthma exacerbations and OCS sparing effects (Haldar et al., 2009; Nair et al., 2009). The landmark DREAM phase 2b trial reaffirmed this relationship and established the important principle of stratified patient selection by a simple, clinically accessible biomarker (the blood eosinophil count) to successfully influence asthma practice (Pavord et al., 2012). In this study, blood eosinophilia emerged as a better predictor of response than sputum eosinophil counts or FeNO.

The subsequent phase 3 trials of mepolizumab (Bel et al., 2014; Chupp et al., 2017; Ortega et al., 2014; MENSA, SIRIUS, and MUSCA) and reslizumab (Castro et al., 2015) confirmed a 50% reduction in exacerbations in patients with severe eosinophilic asthma. They also demonstrated a significant improvement in lung function, symptoms, and quality of life measures, particularly in patients with higher blood eosinophil counts. The SIRIUS trial for mepolizumab found a 50% reduction in baseline OCS dose compared with no reduction in the placebo arm.

Benralizumab targets IL-5Rα on the surface of eosinophils and basophils. Benralizumab differs from mepolizumab and reslizumab because it causes antibody-dependent cell-mediated cytotoxicity leading to apoptosis of eosinophils (Dagher et al., 2022). It eliminates blood eosinophils rapidly, often within 4 h, and reduces sputum eosinophils by at least 90% (Moran et al., 2020). The phase 3 trials of benralizumab in severe asthmatics with blood eosinophils >0.3 × 10^9^ per liter (SIROCCO and CALIMA) decreased the annualized exacerbation rate by up to 50% and increased forced expiratory volume in 1 s (FEV_1_) by 110–160 ml (Bleecker et al., 2016; FitzGerald et al., 2016). The ZONDA study demonstrated a 75% reduction in OCS-dosage compared with 25% in the placebo group (Nair et al., 2017).

There are no head-to-head trials between benralizumab and mepolizumab to compare efficacy. However, a phase 3 clinical trial comparing depemokimab, a subcutaneously administered anti-IL5 with 6 monthly dosing, with either existing mepolizumab or benralizumab treatment is recruiting (GlaxoSmithKline, 2021).

### Anti-IL4Rα treatment

The complementary IL-4 and IL-13 pathways have been recognized as potential therapeutic targets owing to their wide-ranging roles. However, two anti-IL13 monoclonal antibodies, tralokinumab and lebrikizumab, showed limited efficacy in reducing exacerbations in people with high blood eosinophils, FeNO, or serum periostin (Panettieri et al., 2018; Hanania et al., 2016). Further research into both drugs was terminated. There was some improvement in lung function with anti-IL-13 treatment, suggesting that the pathogenesis of exacerbations and reduced lung function may not be the same. Pitrakinra, targeting the α subunit of IL-4R, thereby blocking signaling of IL-4 and IL-13, suffered from similar problems to early mepolizumab trials by testing in unselected asthma populations with no clinical efficacy. Subsequent research showed that it was more effective in specific patients carrying a single nucleotide polymorphism within the IL-4Rα gene, as well as raised blood eosinophils and FeNO (Slager et al., 2012).

Dupilumab is a subcutaneous monoclonal antibody targeting IL-4Rα and has shown consistent efficacy. The phase 3 trial (LIBERTY QUEST) in uncontrolled moderate to severe asthmatics demonstrated a 48% reduction in exacerbations and 130 ml improvement in FEV1 compared with a placebo (Busse et al., 2018). Dupilumab showed a significant improvement in exacerbations in patients with blood eosinophils >0.15 × 10^9^ per liter, but the strength of the effect increased as baseline blood eosinophil count increased. Above 0.3 × 10^9^ per liter, there was a 66% reduction in exacerbations and 220 ml FEV1 improvement. Prespecified subgroup analyses showed that higher baseline FeNO, particularly above 50 ppb, was associated with a larger exacerbation and lung function benefit. The findings were consistent irrespective of whether the patient was having allergic or non-allergic asthma. LIBERTY VENTURE revealed a 70% reduction in OCS maintenance dose in patients treated with dupilumab compared with a 42% decrease in patients treated with a placebo (Rabe et al., 2018).

Additionally, dupilumab is efficacious and licensed for atopic dermatitis and chronic rhinosinusitis with nasal polyposis (Simpson et al., 2017; Bachert et al., 2019). Clinical trials are underway in allergic bronchopulmonary aspergillosis (ABPA; Regeneron Pharmaceuticals, 2020). These other type 2-high diseases frequently coexist with asthma.

### Anti-epithelial cytokine treatment

The alarmins offer the potential to have more wide-ranging effects on downstream cytokines and hematopoietic cells.

Tezepelumab, a subcutaneously administered anti-TSLP monoclonal antibody has been licensed for severe asthma. The phase 3 trial (NAVIGATOR) in patients aged 12–80 yr showed a 56% reduction in exacerbations and 130 ml improvement in FEV1 compared with a placebo (Menzies-Gow et al., 2020). Tezepelumab had the highest efficacy in patients with a type 2-high phenotype. There was a 77% reduction in exacerbations in patients with baseline blood eosinophils >0.45 × 10^9^ per liter. Similarly, there was a 73% reduction in exacerbations in patients with FeNO > 50 ppb. Tezepelumab showed clinical efficacy, albeit less pronounced, in the type 2-low phenotype. There was a 41% reduction in exacerbations in patients with baseline blood eosinophils <0.3 × 10^9^, and a 32% exacerbation reduction in patients with FeNO < 25 ppb. However, the SOURCE study did not show a significant reduction in maintenance OCS between tezepelumab and placebo (Wechsler et al., 2020). The DESTINATION phase 3 extension study over 2 yr showed a continued 58% reduction in annualized exacerbations versus placebo irrespective of baseline biomarker, although results for participants who were both low eosinophil and low FeNO were not presented (Menzies-Gow et al., 2023). Additionally, the CASCADE phase 2 mechanistic study showed that tezepelumab reduced mucous plugging and eosinophilic airway inflammation compared with placebo (Brightling, 2022). An ongoing study is examining the efficacy of tezepelumab in severe chronic rhinosinusitis with nasal polyposis (WAYPOINT; AstraZeneca-Amgen, 2021).

There are also drugs targeting IL-33 (itepekimab) and the IL-33 ST2 receptor (astegolimab). A phase 2 comparing dupilumab, itepekimab, itepekimab/dupilumab, and placebo study showed that itepekimab prevented loss of asthma control compared with a placebo when LABA and then ICS were removed sequentially (Wechsler et al., 2021). However, the effects were not as strong as the dupilumab arm, and the dual biologic arm did not show extra benefit. The lack of additional efficacy with anti-IL33 treatment is probably because of redundancy and the presence of other epithelial cytokine-promoting type 2 inflammation.

Astegolimab was tested in a phase 2 trial with no minimum threshold for blood eosinophils. Astegolimab reduced exacerbations by 43% compared with placebo at the 490 mg dose (Kelsen et al., 2021). There was a comparable positive effect in the purely eosinophil low group.

### Safety of biological agents in asthma

All biologicals carry a similar low risk of injection site reaction (<5%) and a very low risk of hypersensitivity reaction (<0.5%). The most notable side effect of dupilumab was blood hypereosinophilia of unknown significance. This occurs because the transport of eosinophils from the bloodstream is impeded. A blood eosinophil count of 3 × 10^9^ per liter was noted in 1.2% of dupilumab recipients, but these patients were asymptomatic (Wechsler et al., 2022). There are rare case reports of eosinophilic granulomatosis with polyangiitis following dupilumab treatment (Tanaka et al., 2022). The most common side effects of tezepelumab treatment were nasopharyngitis, injection site reaction, upper respiratory infection, and headache.

Susceptibility to parasite infection is a theoretical concern with anti-IL5 and anti-IL-4Rα therapy owing to the central role of type 2 immunity in parasite host defense. Mouse models with eosinophil depletion have generally shown appropriate primary and secondary immune responses to a variety of helminths (Jackson and Pavord, 2023). Mouse models with eosinophil and neutrophil depletion had reduced larval killing, suggesting immune redundancy compensating for the reduced eosinophils. However, a mouse model of IL-4/IL-13 depletion did show reduced gut larval expulsion of *Nippostrongylus brasiliensis* (Oeser et al., 2015). In humans, none of the anti-IL5 monoclonal antibodies have shown a signal of increased parasite infection in clinical trials or real-world data, even in areas where parasitic infection is endemic. Only one study of dupilumab in a pediatric asthma setting showed a small signal of parasite infection, but this is not replicated in other dupilumab studies (Bacharier et al., 2021).

The other main theoretical concern with monoclonal antibodies targeting type 2 immunity is the long-term risk of increased malignancy. Eosinophils are known to infiltrate the microenvironment of multiple tumors, but their role is uncertain. In vitro studies suggest that eosinophils have an antitumorigenic effect through direct cytotoxic actions or indirect effects (Grisaru-Tal et al., 2020). Mouse models demonstrate conflicting evidence with a mixture antitumorigenic and protumorigenic outcomes in the presence (or absence) of eosinophils. In humans, clinical trial and long-term follow-up data have not shown a signal for malignancy beyond what is expected of the normal population. Nonetheless, given the latency of cancer, long-term surveillance registries for asthma monoclonal antibodies are important (Jackson and Pavord, 2023).

### New treatments on the horizon

JAK inhibitors are an attractive small molecule option given the widespread signaling of cytokines and growth factors using JAK-STAT receptors in asthma, including IL-4, IL-5, IL-13, GM-CSF, IFN-α, IFN-β, IFN-γ, and TSLP (Georas et al., 2021). There has already been success in trialing JAK-1 inhibitors in atopic dermatitis, and phase 1/2 studies are underway in asthma in oral and inhaled preparations.

Amlitelimab is a human mAb that binds to the OX40-ligand (OX40L) and offers a potential novel treatment for asthma. OX40L is expressed on antigen-presenting cells, such as DCs, endothelial cells, macrophages, and activated B cells (Lé and Torres, 2022; Sanofi, 2022). OX40 is expressed on regulator and effector T cells. OX40L-positive DCs induce OX40-positive T cell differentiation, including Th2 cells, which promote IL-4 and IL-13 production, as well as Th1 and Th2 pathways. Amlitelimab has already had marked clinical improvement in atopic dermatitis with a good safety profile. A phase 2 trial in asthma is underway (Sanofi, 2022).

Bruton’s tyrosine kinase inhibitors (BTKi) are another class of small molecules already in use for B cell malignancies and are being explored in allergic asthma. BTK is an important kinase for FcεRI signaling in mast cells and basophils, therefore mediating allergic-driven inflammation (Dispenza, 2021). An inhaled BTKi showed more potent inhibition of antigen-induced pulmonary inflammation than inhaled budesonide (Phillips et al., 2016). Rilzabrutinib, an oral BTKi, is currently undergoing a phase 2 trial. Adverse effect burden is a problem with BTKi and, even if efficacy is proven, may limit the use of these drugs in all but the most severe allergic-driven asthma cases.

Atuliflapon is a FLAP inhibitor that reduces 5-lipoxygenase (5-LO) pathway activity and the production of proinflammatory leukotrienes. Following an inflammatory stimulus, 5-LO interacts with 5-lipoxygenase-activating protein (FLAP) on the nuclear membrane, which mediates arachidonic acid transfer to the active site of 5-LO (Pettersen et al., 2019; AstraZeneca, 2022). Atuliflapon is currently in a phase 2 trial comparing efficacy and safety with montelukast, a licensed leukotriene receptor antagonist (AstraZeneca, 2022).

Currently, licensed monoclonal antibodies in asthma are all injected, so there is scope to deliver treatment less invasively. Dexpramipexole is an oral medication originally developed for amyotrophic lateral sclerosis but was incidentally found to significantly reduce the blood eosinophil count. The mechanism of action is thought to be by inhibiting the maturation of promyelocytes into eosinophils in the bone marrow. In the phase 2 EXHALE study, there was an 80% reduction in asthma exacerbations and a non-significant 172-ml increase in pre-bronchodilator FEV1 by week 12 (Siddiqui et al., 2021). Additionally, inhaled versions of IL4Rα and TSLP fragments are in early-stage clinical trials and potentially offer alternative asthma treatment to ICS.

Furthermore, combining biologics targeting different type 2 pathways is being explored by drug companies. This could offer the benefit of having a portfolio of options that could target the mechanisms driving an individual person’s disease—a goal of precision medicine. However, this approach could also potentiate the side effects outlined above.

Beyond type 2 inflammation, novel treatments may emerge by targeting the other treatable traits. Neutrophil extracellular traps (NET) are formed when neutrophils shape a web of DNA and protein-rich fibers to immobilize pathogens (Keir et al., 2021). Evidence of their role in asthma is growing, particularly those with neutrophilic inflammation or persistent airway infection with *Haemophilus influenzae* (Brown et al., 2022). Targeting NETosis with dipeptidyl peptidase-1 (DPP-1) inhibitors is an attractive prospect (Chalmers et al., 2020).

Mucous plugging is an important patient symptom and there is emerging longitudinal computerised tomography evidence that persistent small mucous plugs lead to an increase in air trapping and airway obstruction (Fahy and Dickey, 2010; Tang et al., 2022). Finding new therapeutics that modulate mucous production without affecting host defense is a priority. Charcot-Leyden crystals (CLCs) have emerged as an important target because of their abundance in people with eosinophilic asthma. CLCs are formed as part of a regulated pathway of extracellular trap death by eosinophils (ETosis) from galectin-10 (Ueki et al., 2018). Persson et al. (2019) examined the structure of patient-derived CLCs and determined that crystalline galectin-10 acted as a type 2 adjuvant in mice. Antibodies that dissolved CLC showed potential as a future drug target by reversing this inflammation and reducing bronchial hyperreactivity in mice. Additionally, peptides that disrupt Ca^2+^-triggered membrane fusion in the production of mucin are a promising opportunity to reduce mucous production and have demonstrated efficacy in mice (Lai et al., 2022).

Finally, obesity is a major driver of asthma symptoms and healthcare utilization. Part of the symptom burden is from changes to lung physiology. But mouse models also suggest that obesity alters inflammatory mechanisms by switching type 2 T helper–predominant disease to more severe, less responsive type 17 T helper–predominant disease. This may be related to decreased activity of nuclear receptor peroxisome proliferator-activated receptor-γ (PPARγ; Bapat et al., 2022; Vanderbilt University Medical Center, 2022). Treatment with a small molecule PPARγ agonist has the potential to reverse this inflammatory switch and reawaken therapeutic responsiveness to type 2 biological therapy. Alternatively, the problem could be addressed directly. Weight loss through dietary modification is effective but rarely sustainable. Novel glucagon-like peptide-1 (GLP-1) receptor agonists deliver marked weight loss and may prove to be a useful adjunct in the severe asthma toolbox (Wilding et al., 2021; Vanderbilt University Medical Center, 2022).

### Biomarker-directed approach to treating asthma

Asthma is fraught with misdiagnosis and mistreatment by attributing non-specific symptoms to the label of asthma (Aaron et al., 2017). Given that asthma attacks are usually defined by an acute deterioration in symptoms, patients may receive harmful OCS treatment unnecessarily. Biomarkers offer a useful, objective marker to support diagnosis and guide treatment. This is particularly pertinent in the age of targeted monoclonal antibody therapy. The two most accessible and evidence-based biomarkers to emerge are blood eosinophils and FeNO.

There is emerging evidence that blood eosinophils and FeNO are additive prognostic markers for the risk of future exacerbations (Couillard et al., 2022). Symptom-based severity definitions and treatment algorithms do not capture the future risk of asthma attacks. Indeed, up to 30% of asthma deaths are in people with infrequent symptoms who could be easily misclassified as having “mild asthma.” We advocate that all patients should have these biomarkers checked when making a diagnosis of asthma and repeat it at each exacerbation prior to treatment. Elevated biomarkers can be used to guide the escalation of inhaled treatment accordingly or referred for consideration of biological therapy. A key area of future research will be to identify whether biomarker “hot” patients who do not exacerbate also suffer lung function decline and airway remodeling. These people may benefit from earlier aggressive intervention, for example with biological therapy.

Future research should also delve into the causes and mechanisms of asthma attacks. We know that viral infection is often implicated, but why they do not always lead to an attack and the impact of viral infections on the lower airway immunopathology in type 2-high asthma are not well understood. The MUPPITS-1 study observed heterogeneous nasal transcription modules in viral positive and viral negative asthma attacks in children (Altman et al., 2019). The MUPPITS-2 trial of mepolizumab showed that some children have elevated nasal epithelial non-type 2 inflammatory pathways that continue to drive attacks (Jackson et al., 2022). More research that precisely phenotypes people’s asthma attacks to predict their optimal treatment is needed. We should also question the role of oral steroids in asthma attacks—the ultimate one-size-fits-all treatment—and whether they are effective in biomarker-low exacerbations. In biomarker-high asthma attacks, a clinical trial is already underway to test if benralizumab is more clinically effective than oral steroids (University of Oxford, 2019).

## Conclusion

The field of asthma has undergone a radical transformation over the past 20 yr. Patients with significant unmet needs now have access to highly effective and safe treatments in the form of biological therapies. These treatments yielded from our heightened understanding of type 2 inflammation and its interplay with pathophysiology in asthma. But the early failures of treatments in all-comer asthma patients underscores the important narrative that asthma should be diagnosed and treated in a targeted manner. As new treatments emerge for type 2-high or type 2-low asthma, it is imperative that we persist with the principle of precision medicine.
